# Evaluating community resilience through social media during China’s first post-COVID-19 reopening: insights from machine learning

**DOI:** 10.7189/jogh.15.04315

**Published:** 2025-11-21

**Authors:** Shouchuang Zhang, Lanyue Zhang, Jiayi Weng, Danijela Gasevic, Yuehui Wei, Zefeng Chen, Jun Zhang, Larry Z Liu, Weiyan Jian

**Affiliations:** 1 Department of Health Policy and Management, School of Public Health, Peking University, Beijing, China; 2 School of Mathematics and Statistics, Xi’an Jiaotong University, Xi’an, China; 3 School of Public Health and Preventive Medicine, Monash University, Melbourne, Australia; 4 Department of Industrial and Systems Engineering, University of Washington, Seattle, Washington, USA; 5 MSD R&D (China) Co., Ltd., Beijing, China; 6 Merck & Co., Inc., Rahway, New Jersey, USA; 7 Weill Cornell Medical College, New York, New York, USA; 8 Key Laboratory of Health System Reform and Governance, National Health Commission of China, Beijing, China

## Abstract

**Background:**

In the face of pandemics from infectious diseases, enhancing community resilience is increasingly important. It is, therefore, essential to evaluate community resilience and identify factors that can strengthen it. This study aimed to evaluate community resilience by leveraging a data set comprising user information from Weibo and applying interpretable machine learning (ML) techniques to identify the contributions of various indicators underpinning community resilience.

**Methods:**

This cross-sectional study analysed social media data from December 2022 to January 2023. COVID-19-related user interactions were examined as indicators of community resilience within the context of community response. This study introduced an evaluation framework comprising thirteen indicators. It also described the application of natural language processing (NLP) techniques, the K-means (KM) clustering, a random forest (RF) classifier and SHapley Additive exPlanations (SHAP) to achieve its objectives.

**Results:**

A total of 177 000 Weibo posts were collected for this study. The NLP model demonstrated strong performance in accurately labelling posts, with the area under the curve (AUC) of 0.8862 (95% confidence interval (CI) = 0.8600–0.9102) and accuracy (ACC) of 0.8939 (95% CI = 0.8563–0.9277). This study identified four distinct community resilience levels: low (77.64%), medium-low (9.86%), medium-high (10.55%), and high (1.95%). Further analyses revealed clear regional disparities in community resilience, with higher levels observed in Eastern China. The top five indicators associated with community resilience, as determined by mean SHAP values, were ‘Efficacy of performance altruistic response’ (0.0101), ‘Tangible aid engagement’ (0.0051), ‘Rapid performance of altruism’ (0.0044), ‘Sentiment response associated with recording positive posts’ (0.0036), and ‘Help-seeking response efficacy’ (0.0035).

**Conclusions:**

This study is the first to harness social media data to quantify community resilience in mainland China. Five indicators associated with enhanced community resilience are identified as potential predictors that can inform governmental strategies and strengthen decision-making support for improving health emergency responses.

The Coronavirus Disease 2019 (COVID-19) pandemic affected more than 100 countries, placing immense pressure on global health systems and socioeconomic structures [1,2]. In December 2022, China began transitioning away from its dynamic ‘zero-COVID’ policy and initiated phased re-opening of various country regions [3]. This policy shift resulted in a surge in confirmed cases and a rise in hospitalisation rates [4]. Concurrently, there was a notable increase in community-level demand for antipyretic medications. Between early December 2022 and 12 January 2023, approximately 60 000 COVID-19-related deaths were reported in Chinese health facilities [5]. These developments placed significant strain on medical resources and revealed new challenges for public health systems and residential communities. Given this unique and critical context, we selected the period between December 2022 and January 2023 as the study window. This timeframe captured the earliest stage of reopening, when community responses were most visible, and resilience mechanisms were actively mobilised under sudden and extreme stress [4]. The period also provided a natural experiment to observe resilience, as communities were confronted simultaneously with soaring infection rates, shortages of essential medicines, and overwhelming demands on health services [6].

Social media plays a pivotal role in crisis communication and community response during public health emergencies. It not only strengthens social connections and facilitates resource mobilisation but also provides real-time data on public sentiment and collective behaviour [7]. During COVID-19, platforms such as Weibo, Twitter, and Facebook were widely used by individuals to document experiences, request assistance, and share health-related information [8,9]. These activities both fostered psychological support and shaped coordinated governmental and community responses [10,11]. Weibo, in particular, is the most widely used public microblogging platform in China, with approximately 587 million monthly active users and 257 million daily active users as of September 2024 [12]. During the first wave of infections after reopening, Chinese residents actively shared immediate needs, personal experiences, and practical guidance through Weibo posts [13]. The originality and timeliness of this content provide an unfiltered perspective on how communities adapted and mobilised during the crisis [14]. As such, Weibo data represent a valuable source for analysing the dynamics of community resilience, revealing both behavioural adaptations and structural challenges under extreme public health stress.

Community resilience refers to a community’s capacity to withstand, recover from and adapt to disasters [15], as well as the collective efficacy of its members in addressing associated challenges [16]. Many communities have mitigated the adverse effects of COVID-19 by leveraging support available through social networks, including assistance with mental health and the fostering of mutual aid and shared understanding among residents [17]. Community resilience can be evaluated from various perspectives, such as information exchange, psychological cohesion, collective effectiveness, and social trust [18–20]. A growing body of research suggests that social media data can both offer valuable insights into community resilience and serve as a tool to strengthen it [21–23]. Given the crucial role resilient communities play in responding to public health crises, it is essential to identify key factors influencing community resilience through the analysis of social media data.

Previous researches have provided the theoretical basis for the analysis of community resilience. Norris et al. (2008) proposed one of the most influential multidisciplinary models of community resilience. They conceptualised resilience as a process linking sets of adaptive capacities, specifically economic development, social capital, information and communication, and community competence, to positive adaptation following disasters. Each capacity contributes uniquely: social capital fosters trust and reciprocity, information and communication reduce uncertainty, and community competence ensures effective collective action. This framework highlights resilience as a dynamic networked process rather than a static attribute, emphasising redundancy, robustness, and rapidity as qualities of adaptive capacity [24]. Pfefferbaum et al. (2013) advanced this understanding by developing the Communities Advancing Resilience Toolkit (CART), which operationalises resilience into four domains: connection and caring, resources, transformative potential, and disaster management. These domains underscore the importance of psychosocial support (connection and caring), tangible and intangible assets (resources), the community’s capacity for collective learning and adaptation (transformative potential), and organisational readiness (disaster management). Communities Advancing Resilience Toolkit has been widely applied as a measurement tool, bridging conceptual frameworks and empirical assessment [25]. In addition to these integrative frameworks, classical social theories provide further grounding. Sampson et al. (1997) introduced the theory of collective efficacy, defined as social cohesion among neighbours combined with their willingness to intervene for the common good. This theory demonstrated that beyond mere network density, communities with higher collective efficacy exhibit stronger capacities to regulate behaviour and respond collectively to challenges, making it a central construct in resilience research [26]. The theory of social capital, broadly defined as the resources embedded in social networks and relationships, is a key foundation of community resilience [27]. Scholars typically distinguish between bonding social capital, which provides emotional and material support through strong ties, and bridging social capital, which expands access to information and resources through weaker ties across groups [28,29]. Both forms are critical in crises: bonding capital sustains trust and mutual aid, while bridging capital enables broader coordination and recovery. Finally, the emerging field of crisis informatics underscores the critical role of information flows and communication technologies in disaster contexts. Research in this domain demonstrates how transparent, timely, and widely accessible information supports situational awareness, coordination, and resource matching across formal institutions and informal community networks [30].

A systematic review of more than 80 studies has identified nine recurring elements of community resilience: local knowledge, community networks and relationships, communication, health, governance and leadership, resources, economic investment, preparedness, and mental outlook. Their analysis underscored both the conceptual heterogeneity of resilience and the common elements that consistently emerge across diverse contexts. A key contribution of this review was to highlight that community resilience is best understood as a set of interrelated but distinct components, each of which can be examined and measured separately [31]. Together, previous frameworks converge on a set of interrelated domains: competence and collective action, psychosocial and emotional support, social capital and networks, communication and information flow, and organisational responsiveness. that informed the five dimensions adopted in this study. Therefore, these domains constituted the five key features of community resilience adopted by this study, which were collective efficacy, community emotional support, social capital support, community information sharing, and rapid response performances of community networks.

Machine learning strategies have shown considerable promise in public health investigations, particularly in understanding community responses to the COVID-19 outbreak [32,33]. Machine learning can efficiently process large-scale, unstructured data sets-such as social media posts-enabling the analysis of vast volumes of data to predict epidemic spread, evaluate intervention effectiveness, and monitor public responses and behavioural patterns [34]. Recent studies have also applied ML to assess risks and community resilience in urban areas, offering novel approaches relevant to both urban management and disasters research [35]. Moreover, ML can be used to identify and predict key risk factors affecting health outcomes [36,37]. In the context of public health, ML techniques have thus emerged as powerful tools, providing data-driven insights to support evidence-based decision-making [38,39].

Previous studies have evaluated community resilience through social media data, however, such research has been limited to specific types of communities [21,40]. Furthermore, the application of ML algorithms for assessing community resilience remains underexplored. There is a relative lack of systematic research focused on evaluating community resilience through social media, resulting in the absence of a clear framework for extracting and analysing key indicators using ML techniques. Therefore, the aims of this study were to construct an indicator system for evaluating community resilience based on data set of Weibo users and to employ interpretable ML techniques to identify the contributions of different community resilience indicators.

## METHODS

### Study design and data collection

This study aimed to utilise NLP techniques to categorise COVID-19-related Weibo posts made by mainland Chinese residents during the initial phase following the country’s reopening. The analysis focused on identifying post types, stakeholder behaviours, and the different patterns of responses to such posts. Based on thirteen indicators across five features, the KM clustering method was applied to stratify levels of community resilience. In addition, an ML algorithm (a RF classifier) was used to estimate community resilience levels and determine the relative importance of each indicator. SHapley Additive exPlanations (SHAP) was further used to interpret the model outputs, providing insights into the estimated contributions of individual indicators to community resilience.

Data for the study were obtained from the Sina Yuqingtong big data system (https://www.yqt365.com/), which has signed a data purchase agreement with Sina Weibo. By setting the post location to mainland China, all COVID-19-related Weibo posts with geotags were collected between 8 December 2022 and 7 January 2023. Search parameters were established to target posts containing various expressions paraphrasable as ‘help’ or ‘help-seeking’ in Chinese (Text S1, Table S1–2 in the **Online Supplementary Document**) along with references to conventional COVlD-19 medications that were in short supply at the time. These included specific drugs such as ‘Ibuprofen’, ‘Ambroxol’, and ‘Acetaminophen’, as well as general references to antipyretic and symptomatic treatments commonly used during the outbreak.

The sample size was not determined by prior power calculation but instead reflected the full availability of COVID-19-related posts during the reopening period. This large-scale data set provided sufficient variation and statistical power to support the construction of a robust evaluation framework for community resilience. Representativeness was enhanced by systematically retrieving posts geotagged across all provinces of mainland China, ensuring geographic diversity.

All posts were directly retrieved from the Sina Weibo platform using predefined keywords, and the downloaded data set did not contain missing values for the selected indicators. Accordingly, no imputation procedures were required.

### Analysis of Weibo posts

#### Coding procedure

A standardised data encoding format was developed to enable effective analysis of the help-seeking Weibo posts from the study period. The coding framework included four dimensions: Space, Content, Network and Time (Text S2 and Table S3 in the **Online Supplementary Document**). Each Weibo post was encoded using a binary tagging system: if a post aligned with a specific item within a given category, it was assigned a value of ‘1’; if not, it was assigned a ‘0’. This binary approach allowed for structured and efficient data analysis.

An initial trial coding exercise was conducted prior to applying NLP techniques for full data set labelling. We randomly selected 2000 posts from the final cleaned data set, which were independently coded by several authors using the predefined coding rules [41,42]. This pilot exercise allowed the team to identify coding challenges, discuss discrepancies collectively, and refine the coding framework to ensure clarity and consistency. Coding discrepancies were resolved through consensus discussions within the core coding team, which included SZ (lead), LZ (support), JW (support), YW (support), and ZC (support). Other co-authors (DG, JZ, LL, and WJ) provided conceptual and methodological support throughout the process. To further ensure consistency, team members reviewed and cross-checked the coding results, maintaining an inter-coder agreement of over 90% [43]. After the coding framework was stabilized, we constructed a subset of 10 000 posts randomly selected from the final cleaned data set. To further ensure the rigor and reliability of labelling process, two authors (SZ and LZ) manually coded 10 000 posts. This final labelled data set of 10 000 posts was subsequently used for training and evaluating the text classification model [41,42].

#### Text classification

To perform text classification, we employed the BERT-based Chinese language model, a transformer architecture developed by Google, which enables efficient processing and classification of Chinese text using NLP techniques [44]. BERT-based Chinese language model was selected because it represents the state-of-the-art in Chinese NLP, capturing contextual and semantic dependencies more effectively than traditional models such as bag-of-words. This was particularly important for accurately classifying heterogeneous Weibo posts, which often contain colloquial, context-dependent expressions [45]. Text classification training was conducted for the top 16 items across the coding framework, excluding the Time dimension. A 5-fold cross-validation approach was adopted, with 80% of the labelled data allocated for training and the remaining 20% reserved for testing the model’s performance [46].

The cross-entropy loss function (nn.CrossEntropyLoss) was used as the loss function for model optimisation. The model’s parameters included 2 Epochs, a Batch Size of 4, a Max Segment Length of 512, and a Learning Rate of 1 × 10^−5^. Model performance was evaluated using the AUC and ACC scores, with values approaching 1 indicating strong classification performance. To determine the final classification labels, a soft voting ensemble method was applied. In addition to AUC and ACC, we also computed Precision, Recall, and Fl-score with 95% CI to comprehensively assess classification performance. Higher values of these metrics indicate better predictive accuracy and model robustness [47].

### Evaluating community resilience and identifying indicators importance

#### Operationalisation of indicators

To comprehensively assess community resilience, we identified 13 indicators grouped under these five features. The operational definitions of the thirteen indicators are summarised in **Table 1**.

**Table 1:** Measurements of thirteen indicators grouped under five features

Features	Indicators	Explanatory
Collective efficacy	Help-seeking response efficacy (X_1_)	Community residents receive different stakeholders (*e.g*. community-level, governmental or market-based) responses and solve problems through social media. Scoring rang: receive community-level responses = ‘2’; receive other (*e.g*. governmental or market-based) responses = ‘1’; no responses = ‘0’
	Efficacy of performance of altruistic response (X_2_)	
Community emotional support	Sentiment response associated with help-seeking (X_3_)	Community residents receive emotional expression (*e.g*. positive or negative) at the community-level on social media. Scoring range: positive = ‘1’; none = ‘0’; negative = ‘−1’
	Sentiment response associated with altruistic posts (X_4_)	
	Sentiment response to the sharing of official information (X_5_)	
	Sentiment response associated with recording positive posts (X_6_)	
	Sentiment response associated with recording negative posts (X_7_)	
Social capital support	Tangible aid engagement (X_8_)	Community residents receive social capital support at the community-level on social media. Scoring rang: tangible support = ‘2’; intangible support = ‘1’; none = ‘0’
	Intangible aid engagement (X_9_)	
Community information sharing	Interaction relating to repost/share official information (X_10_)	Official information receive community-level responses on social media across different time intervals. Scoring rang: receive community-level responses = ‘1’; no responses = ‘0’
Rapid response performances of community networks	Rapid aid reaction (X_11_)	Community residents receive rapid responses through social media. Scoring rang: receive responses within 1 h = ‘1’; others = ‘0’.
	Rapid performance of altruism (X_12_)	
	Rapid sharing of official information as a reaction (X_13_)	

Collective efficacy, defined as residents’ shared beliefs in their collective capacity to achieve common goals, is a foundational element of community resilience [48,49]. During the COVID-19 pandemic, the widespread use of social media platforms by residents to seek help or assistance can be interpreted as a form of collective behaviour aimed at mobilising community support to address the health crisis.

Community emotional support became essential as the pandemic posed significant psychological and social challenges to individuals, families and communities [50,51]. While negative emotions, such as fear and anxiety were common, the presence and expression of positive emotions played a crucial role in mitigating the adverse psychological effects of the crisis [52]. Emotional support was operationalised through posts that included expressions of gratitude, encouragement, or empathy, either when users received help or actively offered support through sharing, commenting, or reposting related content.

Social capital support is recognised as one of the strongest predictors of perceived community resilience [27,53]. It encompasses both tangible resources (physical items, services, financial support) and intangible resources (emotional, psychological or informational support) that collectively reinforce the social infrastructure of communities [27,29,54]. In this study, posts offering or requesting such resources were coded as indicators of social capital support.

Community information support refers to the use of social media to share reliable, useful, or authoritative information during crises. Community information sharing and support promote information exchange and trust among community members [55], which are key components of community resilience [56]. Posts containing official updates or practical guidance were included as indicators of this feature.

Rapid response performances of community networks relate to the timeliness of interactions following help-seeking or information posts. Social media platforms have a greater advantage in communication speed than traditional media [57]. When community residents post help-seeking posts, altruistic behaviour posts or share effective information, they can trigger timely interactions among users, which is an important feature for responding to health crises. The ability of users to respond within a short window, particularly within one hour, to COVID-19-related posts reflects a community’s real-time coordination capacity. However, compared with other types of information, health emergency information takes longer to be responded to by the public [58], yet the median speed of social media information dissemination is less than one hour [59], making rapid responsiveness a critical component of digital-era community resilience.

#### Clustering

Clustering analysis is a fundamental method for uncovering latent patterns in the data by grouping data objects into multiple clusters based on the intrinsic similarities in measurement or perception [60]. Among clustering techniques, KM clustering is one of the widely used partitioning methods, owing to its simplicity, computational efficiency and ease of implementation [61]. The KM clustering algorithm divides data samples into distinct clusters based on distance metrics. It seeks a partitioning that minimises the squared error between the cluster centroid and the data points within that cluster [62]. The KM clustering algorithm is extensively applied in fields such as computer vision and diabetes diagnosis [63,64]. It is often employed as a preprocessing step for more advanced models to provide an initial configuration.

In this study, KM was selected because it performs reliably on large-scale, high-dimensional data sets such as ours (177 000 posts ×13 indicators) and produces partitions that are both interpretable and replicable. Other approaches, such as hierarchical clustering and density-based methods, have important merits but also limitations for large-scale social data. Hierarchical clustering can generate informative dendrograms and does not require pre-specifying the number of clusters; however, it is computationally expensive for very large data sets and sensitive to linkage criteria, which may lead to unstable results when the sample size is substantial [65]. Density-based algorithms, such as DBSCAN, are effective at identifying arbitrarily shaped clusters and detecting outliers, but their performance depends heavily on parameter tuning and they often struggle with data characterised by heterogeneous densities across clusters [66]. Given these considerations, KM offers a pragmatic balance between scalability, interpretability, and empirical robustness for the present study. Therefore, this study employed the KM algorithms to achieve a stratified classification of community resilience levels.

#### Community resilience levels evaluation

Grid cells within the same cluster were treated as having identical community resilience levels, based on the clustering module’s ability to group cells with similar resilience characteristics [35]. To interpret these clustering results, we employed an explainable classification model, the RF classifier with strong interpretability and performance [67]. Random forest is a non-parametric ensemble method that can model nonlinear relationships and high-order interactions, making it well-suited to complex social data. Moreover, RF is robust to overfitting and does not rely on strict distributional assumptions, which increases reliability in applied settings [68]. This classifier, implemented using scikit-learn, used the resilience-related indicator matrix as input, with the clustering outcomes serving as labels [68]. By analysing the importance of individual resilience indicators in relation to the clustering results, the model provided insights into how these indicators influence the evaluation of community resilience levels. The methodological steps were as follows:

1. Normalisation of indicators: the Min-Max Scaler method was used to normalise all community resilience-related indicators and scale their values to the range of (0–1) to reduce the impact of dimensions and units between different indicators and ensure that subsequent analyses are comparable [69,70].

2. Cluster-level characterisation: the mean value of each indicator was computed for each cluster to characterise the resilience profiles of that cluster.

3. Computation of the aggregated resilience values: a weighted sum approach was used to compute a composite resilience score for each cluster. The weights were derived from the feature importance calculated by the RF model, and applied to the corresponding mean indicator values.

4. Ranking and determining community resilience levels: the comprehensive community resilience value was converted into specific resilience levels through ranking, and clear resilience levels were assigned to different regions to form an intuitive grading system.

#### Model interpretation

We evaluated the importance of community resilience indicators in the model’s prediction using SHAP, a technique derived from cooperative game theory that has been widely applied in ML research to interpret complex models [36,71,72]. SHapley Additive exPlanations has the advantage of global predictability, showing the contribution of each variable to the target outcome [73]. In this study, SHAP values were computed to explain the relative contributions of each resilience indicator [74].

### Statistical analysis

We adopted quantitative descriptive analysis to report the general features and content of the included posts. The characteristics of Weibo posts were calculated using counts (n) and percentages (%). The silhouette coefficient was used to evaluate the effectiveness of KM clustering. The metric ranges between −1 and 1, with higher values indicating a more accurate and reliable clustering result [75]. In addition, the within-cluster sum of squares (WCSS) was examined, and the elbow method was applied to identify the optimal number of clusters [76]. Data analyses were conducted in Python version 3.8 (Python Software Foundation, Wilmington, DE, USA). Two-sided *P* < 0.05 was considered statistically significant. This study has been reported in line with the REFORMS consensus criteria [77].

**Figure 1:**
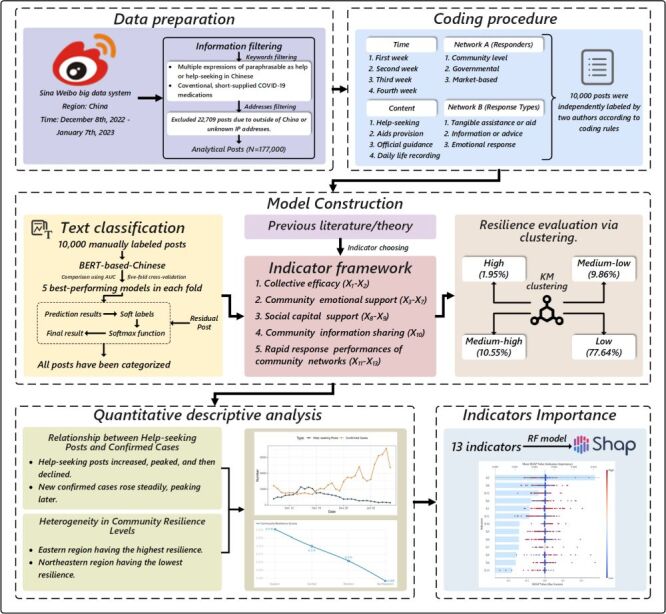
The overall flowchart of this study. X_1_ – help-seeking response efficacy, X_2_ – efficacy of performance of altruistic response, X_3_ – sentiment response associated with help-seeking, X_4_ – sentiment response associated with altruistic posts, X_5_ – sentiment response to the sharing of official information, X_6_ – sentiment response associated with recording positive posts, X_7_ – sentiment response associated with recording negative posts, X_8_ – tangible aid engagement, X_9_ – intangible aid engagement, X_10_ – interaction relating to repost/share official information, X_11_ – rapid aid reaction, X_12_ – rapid performance of altruism, X_13_ – rapid sharing of official information as a reaction.

We conducted the workflow process for this study (**Figure 1**) and the detailed description of the analysis plan (Text S3 in the **Online Supplementary Document**).

### Sensitivity analysis

To evaluate the robustness of feature-importance rankings, we conducted a sensitivity analysis using eXtreme Gradient Boosting (XGBoost), a widely applied ensemble algorithm based on gradient-boosted decision trees [78]. Unlike RF, which constructs multiple trees in parallel through bootstrap aggregation and then averages their predictions, XGBoost builds trees sequentially in a boosting framework. At each iteration, new trees are added to correct the residual errors of the ensemble, and the model is optimised using gradient descent on a differentiable loss function [79]. This procedure enables XGBoost to capture complex nonlinear interactions among features and to achieve high predictive accuracy. In addition, XGBoost incorporates regularisation terms (L1 and L2 penalties) to reduce overfitting and supports advanced techniques such as shrinkage, feature subsampling, and column sampling, which enhance both stability and computational efficiency [80].

We selected XGBoost for comparison because it belongs to the same family of tree-based models as RF and thus allows for consistent SHAP value interpretation, but its different learning strategy (boosting *vs*. bagging) provides an independent robustness check. Specifically, if indicator importance rankings remain stable across these two distinct ensemble approaches, it indicates that the results are robust.

## RESULTS

### Basic data description

By searching for predefined keywords, we initially retrieved 199 790 posts from the Sina Weibo platform. After excluding posts originating from Internet Protocol (IP) addresses outside of mainland China, our final data set comprised 177 000 posts, spanning the period from 8 December, 2022 to 7 January, 2023 (Figure S1 in the **Online Supplementary Document**).

### Performance of NLP model

The model achieved a mean AUC of 0.8862 (95% CI = 0.8600–0.9102), demonstrating a good fit, and a mean ACC of 0.8939 (95% CI = 0.8563–0.9277), indicating an accuracy of 89.39% in labelling posts. This optimal model was then used to categorise the remaining posts. The performance of BERT-based-Chinese ML model on sample data are provided (Table S4 in the **Online Supplementary Document**).

### Sample characteristics

The analysis was performed on a total of 177 000 posts. Among these, 48 425 were categorised as help-seeking posts, 37 180 as aid-provision posts; 16 893 as reposts of official guidance, and 126 195 as daily life recordings (**Table 2**). It is important to note that these categories were not mutually exclusive, as a single post could simultaneously include elements of help-seeking and daily life documentation.

**Table 2:** Sample characteristics

Dimensions	Total, n (%), n = 177 000
**Content**	
Help-seeking	
*Medicine-seeking*	45 619 (25.77)
*Targeting others*	2806 (1.59)
Aid provision	
*Medicine donations*	19 299 (10.90)
*Offering suggestions*	17 881 (10.10)
Official guidance	16 893 (9.54)
Daily life recording	
*Positive*	80 252 (45.34)
*Negative*	45 943 (25.96)
**Network A-Responders**	
Responders	
*Community level*	89 205 (50.40)
*Governmental*	653 (0.37)
*Market-based*	10 849 (6.13)
**Network B-Response types**	
Tangible assistance or aid	
*Community-based donations*	30 808 (17.41)
*Medical suppliers*	2276 (1.29)
Information or advice	
*Guidance regarding medication or home remedies*	18 131 (10.24)
*Information regarding purchasing*	7372 (4.16)
Emotional response	
*Positive*	39 443 (22.28)
*Negative*	8760 (4.95)
**Time**	
First week	57 670 (32.58)
Second week	58 746 (33.19)
Third week	35 337 (19.96)
Fourth week	25 247 (14.26)

A substantial portion of the help-seeking posts, 45 619 out of 48 425 (94.21%) explicitly requested medications, highlighting the acute demand for medical resources during this period. Concurrently, 37 180 (21.00%) posts were identified as aid-provision messages. Of these, 19 299 (constituting over 10%) were related to the donation of medications, further highlighting the critical health concerns surrounding medication access following the ending of the ‘Zero-COVID’ policy.

From the perspective of different responders responding to help-seeking posts, community level replies accounted for 50.40%, demonstrating the pivotal role of communities in the overall assistance system. Among the tangible support provided by residents, mutual aid in medications at the community level represented 17.41%, further emphasising the community’s substantial assistance during the pandemic. Additionally, were 39 443 out of 48 203 (81.83%) responses elicited positive emotional feedback, suggesting that mutual aid among residents helped mitigate the prevailing negative social atmosphere during the pandemic.

Regarding the time distribution, 57 670 posts (32.58%) were published during the first week of the observation period. This was followed by a slight increase in the number of posts during the second week, after which the number of posts gradually declined over the subsequent two weeks.

### Relationship between help-seeking posts and confirmed cases

**Figure 2:**
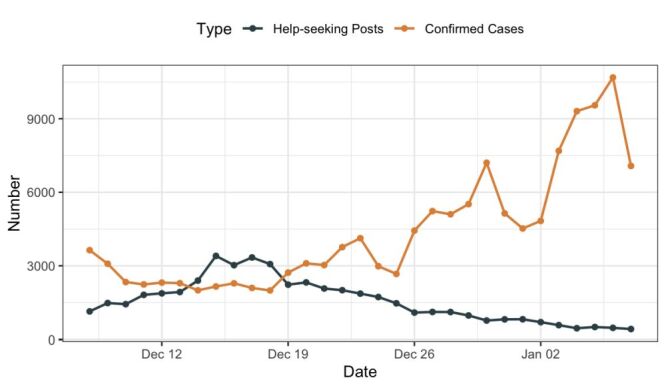
The trend of number of daily confirmed COVID-19 cases and help-seeking posts. *The daily ‘number of newly confirmed COVID-19 cases’ data comes from the Chinese Center for Disease Control and Prevention. †The statistical scope is mainland China.

Over the observation period, the number of help-seeking posts initially exhibited a fluctuating upward trend, reaching a pronounced peak before gradually declining (**Figure 2**). In contrast, the daily number of newly confirmed COVID-19 cases showed a sustained upward trajectory, with its peak occurring noticeably later than that of help-seeking posts. The period from 15 to 18 December marked the peak in help-seeking activity. Following this period, the number of help-seeking posts declined, while the number of confirmed cases surged. Our analysis shows that peaks in help-seeking posts on Weibo preceded the first major peak of confirmed COVID-19 cases by approximately 8 days, the second by 15 days, and the third by 22 days.

### Classification standard of community resilience

We performed KM clustering analysis to classify community resilience into four levels (**Table 3**), and tested its significance. The resulting silhouette coefficient of K = 4 was 0.7003, which was higher than that obtained for alternative cluster numbers K = 3 (0.6894), K = 5 (0.6968), K = 6 (0.6723), and K = 7 (0.6761), indicating that the four-cluster solution provided the most robust classification performance. The scatter plot demonstrates that the four clusters were generally compact and well separated, with most points concentrated in their assigned cluster regions, and the cluster shapes appeared approximately spherical (Figure S2 in the **Online Supplementary Document**). Furthermore, the elbow method based on WCSS showed a clear inflection at K = 4, after which further increases in K only marginally reduced WCSS (Figure S3 in the **Online Supplementary Document**). The classification results show that 137 430 (77.64%) posts were associated with low community resilience, 17 448 (9.86%) with medium-low, 18 678 (10.55%) with medium-high, and 3444 (1.95%) with high resilience levels.

**Table 3:** Classification of community resilience levels

Classification level	No. of posts (n)	Percentage of the total sample (%)
High	3444	1.95
Medium-high	18 678	10.55
Medium-low	17 448	9.86
Low	137 430	77.64

### Results of heterogeneity in community resilience levels

The results indicate notable regional disparities: the Eastern region exhibited the highest levels of community resilience, while the Northeastern region showed the lowest (Figure S4 and Table S5 in the **Online Supplementary Document**).

### Indicators importance

**Figure 3:**
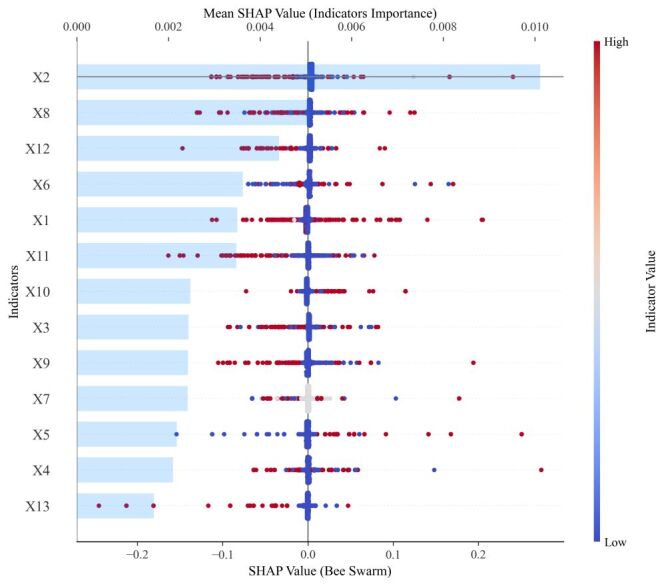
SHAP overall dependency plot. X_1_ – help-seeking response efficacy, X_2_ – efficacy of performance of altruistic response, X_3_ – sentiment response associated with help-seeking, X_4_ – sentiment response associated with altruistic posts, X_5_ – sentiment response to the sharing of official information, X_6_ – sentiment response associated with recording positive posts, X_7_ – sentiment response associated with recording negative posts, X_8_ – tangible aid engagement, X_9_ – intangible aid engagement, X_10_ – interaction relating to repost/share official information, X_11_ – rapid aid reaction, X_12_ – rapid performance of altruism, X_13_ – rapid sharing of official information as a reaction.

The top five indicators contributing most to the prediction of community resilience were as follows (**Figure 3**): ‘Efficacy of performance altruistic response’ (X2) (mean SHAP value, 0.0101), ‘Tangible aid engagement (X8)’ (mean SHAP value, 0.0051), ‘Rapid performance of altruism (X12)’ (mean SHAP value, 0.0044), ‘Sentiment response associated with recording positive posts (X6)’ (mean SHAP value, 0.0036), and ‘Help-seeking response efficacy (X1)’ (mean SHAP value, 0.0035). The directional information displayed in the beeswarm plot indicates that nearly all indicators contribute positively to the enhancement of community resilience. For instance, a higher level of ‘Help-seeking response efficacy (X1)’ was associated with an increased likelihood of promoting community resilience. Details of each indicator’s mean SHAP value are provided (Table S6 in the **Online Supplementary Document**).

### Results of sensitivity analysis

The sensitivity analysis using XGBoost produced feature-importance rankings that were broadly consistent with those obtained from the RF model. Across both algorithms, the top-ranked indicators showed substantial overlap: three indicators (X1, X12, and X2) appeared in the top-5 sets of both models. Although some indicators exhibited modest shifts in their relative positions, the overall pattern of importance remained stable (Table S7 in the **Online Supplementary Document**).

## DISCUSSION

This study employed ML techniques to assess community resilience using social media data, developing an evaluation framework comprising thirteen indicators across five key features. KM clustering was used to stratify community resilience levels, while a RF classifier helped quantify resilience, and determine the contribution of each indicator. SHAP values were used to interpret the model’s predictions. The top five most important indicators associated with community resilience were: ‘Efficacy of performance altruistic response’, ‘Tangible aid engagement’, ‘Rapid performance of altruism’, ‘Sentiment response associated with recording positive posts’, and ‘Help-seeking response efficacy’. These findings underscore the potential of ML algorithms in assessing community resilience and offer valuable insights for government health policy and preparedness strategies in response to future public health crises.

It is important to clarify that social media data were not treated as simple proxies of resilience based on post volume or sentiment alone. While platforms like Weibo can also amplify anxiety or misinformation [81], our approach was to operationalise resilience through 13 indicators grouped into five theoretically grounded features. This design enabled us to capture structural and functional aspects of resilience, such as collective efficacy, information sharing, and network responsiveness, beyond emotional expression or activity levels. At the same time, growing evidence shows that community resilience can indeed be assessed through social media data, which provide valuable insights into dynamic changes in community behaviours and attitudes, as well as into weak links in resilience-building [21–23]. By leveraging such data, researchers and policymakers can better understand how communities respond during public health crises and design more effective and targeted intervention strategies. In this way, the present analysis distinguishes resilience from transient emotional dynamics while also demonstrating the potential of social media data to support resilience assessment and policy development.

The performance of the BERT-based Chinese language model warrants further consideration. The classifier achieved a mean AUC of 0.8862, indicating a high probability of correctly distinguishing between posts belonging to different categories, and a mean accuracy of 0.8939, meaning that nearly nine out of ten posts were correctly classified compared with the human-coded gold standard. These values suggest that the model is well suited to handle the heterogeneity of Weibo texts, including colloquial language and context-dependent expressions, thereby ensuring the reliability of subsequent analyses based on these classifications. At the same time, it is important to recognise the limitations of these metrics: accuracy may be inflated in imbalanced classes, and AUC, while reflecting discrimination ability, does not capture errors related to semantic nuance or cultural context [82]. Future work could therefore complement these quantitative measures with qualitative error analysis [83], for example by manually reviewing misclassified posts, to identify systematic weaknesses such as difficulties in interpreting colloquial expressions, sarcasm, or overlapping categories. Such an approach would provide deeper insights into model behaviour and further enhance the robustness of text classification in resilience research [84].

This study also observed a temporal discrepancy between the rise in confirmed COVID-19 cases and the surge in help-seeking posts on Weibo. Specifically, help-seeking posts on Weibo peaked eight days before the first surge in confirmed cases, 15 days before the second, and 22 days before the third and largest peak. Evidence from other contexts supports this observation: a retrospective analysis of US Twitter data found that social media signals anticipated case surges by approximately 16 days compared to media or Google Trends [85], and a case study of the US and Canada similarly showed that epidemic waves could be detected on social media several days to two weeks earlier than in official surveillance data [86]. Furthermore, institutional systems such as HealthMap and Global Public Health Intelligence Network (GPHIN) have historically identified outbreaks like SARS and Ebola ahead of formal health reporting by leveraging digital and media sources [87,88]. Previous research has suggested that there may be mutual predictive relationship between the epidemic progression and help-seeking behaviour [89] and that the number of help-seeking posts can serve as an early warning signal for COVID-19 outbreaks [42,90]. There is a time lag between residents becoming infected with COVID-19 and receiving a formal diagnosis. During this period, many residents turned to social media to seek assistance. As such, help-seeking posts may reveal early signs of virus transmission, providing valuable supplementary data for epidemic surveillance [42]. During the early stages of the pandemic, the public’s perception of infection risk often precedes official case reporting, resulting in a surge in help-seeking activity. Prior studies have highlighted that, in the early stages of a health crisis, help-seeking messages can reliably predict impending disease outbreaks [91]. These findings highlight the potential for governments and policymakers to harness social media data to anticipate emerging public health threats and to design and implement timely intervention and preparedness strategies at the community level.

We observed marked regional differences in the levels of community resilience levels across China. The Eastern region exhibited higher resilience compared to the Central, Western, and Northeastern regions. This finding suggests that economically developed areas tend to demonstrate greater community resilience during public health emergencies. The higher resilience in the Eastern region may be attributed to its relative advantages in structural metrics, such as economic strength, social capital, health care resources, and Internet penetration, all of which facilitate more effective community responses in times of crisis [92]. Furthermore, cities in Eastern China generally have higher population densities and larger urban scales [93], resulting in higher demand for essential resources, particularly medical supplies, during pandemic outbreaks. In this study, we decomposed community resilience into five operational features with thirteen indicators, which can capture behavioural and coordination processes rather than structural stocks alone. These process-oriented indicators provide real-time insights into how communities mobilise under stress and allow the identification of specific weaknesses (*e.g*. strong information diffusion but slower community response) even within resource-rich regions. Because the indicators are feature-specific and time-stamped, they provide early operational signals during health crises, for instance, detecting surges in help-seeking or delays in response, thereby identifying emerging pressures before they are reflected in conventional structural metrics. Therefore, decision-makers can pinpoint which capacity is weakest and direct resources accordingly, such as volunteer coordination, hotline staffing, or official messaging cadence. Thus, our framework adds value to conventional socioeconomic measures by identifying dimension-specific variations in adaptive mechanisms within distinct regions.

Beyond economic resources, regional heterogeneity in resilience may also be shaped by political culture, information governance, and digital engagement. Eastern provinces and large coastal cities have a longer history of administrative modernisation and neighbourhood-level coordination, which enables more responsive communication between residents and local authorities [94]. In these regions, official and community accounts are more active in publishing timely guidance, addressing rumours, and coordinating online responses, thereby reinforcing information-sharing and collective efficacy signals [95]. The patterns of digital participation and the resources endowment possessed in different regions are also different. The East has higher internet penetration, stronger digital literacy, and denser networks of verified organisations, hospitals, and charities [96]. These conditions allow help-seeking posts to be matched more quickly with practical responses, which strengthens indicators of rapid response and social capital support. Together, these factors suggest that observed differences in community resilience across regions are not only a reflection of structural affluence but also the product of more responsive governance practices and more mature forms of digital engagement.

Previous research has identified collective efficacy as a key predictor of a community’s ability to manage crises and achieve collective goals [18,26,48,97]. Our findings provide additional evidence that collective actions taken by community residents to resist external risks are integral to enhancing community resilience. Following the initial reopening period in China, residents actively used social media platforms to seek assistance from others within their communities. Community-level responses were found to lead to the provision of effective forms of support, such as the supply of drugs and useful information. Concurrently, there were altruistic posts by some residents offering free help, including the sharing of surplus medicines and food. This display of community cohesion mirrors not just the network of interpersonal connections, but also the trust and reliance of residents on their community [98]. In the context of public health emergencies, such coordinated community action strengthens collective efficacy. Essential factor in building and sustaining community resilience.

Our study also finds that community resilience is further reinforced through the tangible support derived from social capital. After the Chinese government announced its comprehensive opening-up policy, there was a rapid increase in residents’ demand for standard COVID-19 medications, such as Ibuprofen and Acetaminophen, particularly for fever treatment. In response to this emerging public health crisis, community members offered surplus medications and essential supplies within their capacity to help neighbours in overcoming challenges. Empirical studies have demonstrated that tangible social support positively influences individuals’ ability to come with risks, reduces symptoms of depression, and improves access to health care services [99,100]. Overall, our findings support the conclusion that tangible support associated with social capital plays a crucial role in promoting residents’ physical and mental health [101,102]. This form of assistance addresses not only the residents’ immediate needs for scarce medications and living supplies, heavily sought after on social media, but also helps alleviate the tension and anxiety during such critical periods.

Previous studies have shown that severe COVID-19 infections waves typically trigger significant shifts in public sentiment, with negative emotions predominantly emerging alongside infection peaks [103,104]. However, we observed the high level of positive emotional expression during the infection wave peak following the end of ‘zero-COVID’ policy. It should also be highlighted that, during emergent public health crises, residents’ positive emotional reactions to community initiatives are correlated with increased community resilience, a finding consistent with existing empirical research [17,105]. Positive content shared by community members on social media can inspire others and strengthen collective in the community’s crisis response. This demonstrates how positive emotional feedback can mitigate the negative psychological impact of crises [52], providing strong evidence for encouraging community members to engage in supportive and optimistic communication during disasters. Such positive interactions help restore confidence and rebuild collective trust in communities’ ability to effectively respond to crises.

Our research indicates that residents actively posted altruistic information on social media and received prompt responses and practical assistance from others, playing an important role in enhancing community resilience. During public health emergencies, residents used social media to promote volunteer services, distribute relief supplies, and share of disaster relief information, enabling residents in need to quickly find and receive help [106]. This rapid interaction not only met residents’ immediate needs, but also enhanced trust and cohesion within the community [107]. The rapid responsiveness of this altruistic behaviour comes from the widespread use of social media, which shortens the time for information dissemination within the community and improves the efficiency of residents in obtaining help [108]. This timely assistance mechanism enables communities to mobilise resources and coordinate efforts more effectively during crises or resource scarcity, reducing anxiety and uncertainty caused by delays and substantially strengthening the communities’ overall response capacity and resilience.

### Public health significance

Our study employed explainable ML to assess community resilience during health crises and to identify key factors influencing it. The findings offer valuable insights for policy recommendations aimed at that enhancing community resilience. To better withstand external uncertainties and risks, community residents should be empowered to effectively use social media for seeking assistance, improve their digital literacy, and engage more actively in online interactions. Moreover, fostering collective resilience through mutual aid, tangible social support and positive emotional encouragement can significantly strengthen communities. In health crises, the ability of community members to collaborate and address challenges via social media enhances the overall resilience of the community.

### Limitations and future research

The current study has several limitations that should be acknowledged. First, relying solely on the data from the Weibo social media platform may have led to omissions, limiting the comprehensiveness of our data set. The representativeness of the sample is also constrained by the demographic and behavioural characteristics of Weibo users, meaning that groups with limited digital access (*e.g*. older adults) may be underrepresented. Moreover, social media data are subject to multiple biases: selection bias, since active posters may differ systematically from silent users; content moderation and censorship, which can remove or amplify certain information; and inauthentic activity, including bots or coordinated accounts. Future research would benefit from integrating data from multiple social media sources to provide a more comprehensive assessment of community resilience. Second, there is currently no unified framework, either domestically or internationally, for selecting resilience indicators derived from social media data. In this study, we operationalised thirteen indicators across five features based on theoretical foundations and prior literature, but the process inevitably involved a degree of subjectivity. The absence of standardised criteria raises challenges for comparability across studies, replication in different cultural or platform contexts, and the cumulative development of knowledge. Future work should therefore prioritise the development of transparent reporting guidelines, consensus-based indicator sets, and validation strategies that triangulate social media-based measures with traditional data sources such as surveys and administrative records to enhance reliability, comparability, and external validity. Third, while the ML models employed in the study performed well, some biases were observed in their outputs, though these remained within acceptable scientific and statistical limits. To enhance the robustness of results, future research should focus on developing improved algorithms tailored specifically to evaluating community resilience. Fourth, resilience indicators were measured at a single point in time, which allows only for cross-sectional associations and prevents us from capturing temporal dynamics or trajectories of resilience. While the reopening period provided a unique and analytically meaningful snapshot of community resilience, the lack of longitudinal data limits our ability to examine how resilience evolves across different stages of a public health crisis. Future studies should extend this framework to multiple time periods and integrate data from diverse contexts to better understand resilience as a dynamic and evolving process. Fifth, while SHAP values offered interpretability by ranking the relative contribution of indicators to resilience classification, they do not establish causal relationships. Therefore, the findings should be interpreted as descriptive associations rather than causal effects. Future research could integrate causal inference frameworks, such as longitudinal designs, instrumental variable approaches, or structural equation modelling, to more rigorously examine the underlying causal mechanisms of community resilience.

## CONCLUSIONS

The COVID-19 pandemic represented an unprecedented global public health crisis. In this study, we employed ML models to analyse social media data and assess community resilience among Chinese residents during the pandemic. Our findings highlighted notable disparities in resilience across mainland China, with economically developed regions demonstrating higher levels of community resilience. We also identified five key indicators that significantly influence community resilience. This study is the first to harness social media data to quantitatively evaluate community resilience in mainland China based on residents’ interactive behaviours. As such, it provides a valuable reference to support governmental efforts in designing policy tools and administrative strategies aimed at strengthening community resilience.

## Additional material


Online Supplementary Document
