# Supplementary material for: Evaluating community resilience through social media during China’s first post-COVID-19 reopening: insights from machine learning
Source: J Glob Health. 2025 Nov 21;15:04315. doi: 10.7189/jogh.15.04315 (PMC12635790; doi:10.7189/jogh.15.04315)
Supplement: Online Supplementary Document [file jogh-15-04315-s001.pdf]

Supplement to: Zhang S, Zhang L, Weng J, Gasevic D, Wei Y, Chen Z, Zhang J, Liu LZ, Jian W. Evaluating community resilience through social media during China's first post-COVID-19 reopening: insights from machine learning. J Glob Health. 2025;15:04315.

Text S1. Keyword List for Weibo Post Search in English (Table S1) and Mandarin Chinese (Table S2)
Text S2. Coding Analysis Framework for Weibo Posts
Text S3. Analysis Plan

Table S1. English Keywords
Table S2. Chinese Keywords
Table S3. Coding Analysis Framework for Weibo Posts
Table S4. Performance Evaluation of Each Text Classification Task
Table S5. Regional Distribution of Mainland China
Table S6. Mean SHAP Value of Indicators
Table S7. Results of Sensitivity Analysis

Figure S1. Flowchart of dataset combination
Figure S2. PCA-based Two-dimensional Visualization of K=4 Clusters **Figure S3**. Elbow Method for Determining the Optimal Number of Clusters **Figure S4.** Community resilience scores across four regions.

References

Text S1

Keyword List for Weibo Post Search in English (Table S1) and Mandarin Chinese (Table S2)

This table provides the complete list of keywords used to identify relevant Weibo posts related to help-seeking and drug-sharing during the COVID-19 pandemic in Mainland China. The keywords are categorized into two groups:

1) ***Help-related terms:*** Words or phrases used to identify posts requesting or offering assistance. Terms within this group were connected by the logical (Boolean) operator OR.
2) ***Medicine-related terms:*** Names of conventional, shortage-prone COVID-19 drugs mentioned in the posts. This encompassed specific medicines listed in the Home Treatment Guidelines for COVID-19 Infected Individuals issued by the National Health Commission of China. These medications were officially recommended for managing symptoms in COVID-19 patients under home isolation. Terms within this group were connected by the logical operator OR.

The relationship between the three groups was defined as follows: a post must have included at least one term from the Help-related group AND the Medicine-related group to be included in the search results.

The keywords are presented in both English (**Table S1**) and Chinese (**Table S2**). Given that the original Weibo posts were in Chinese, our keyword search inherently involved semantic mapping across languages. While this study employs English descriptors such as “help”, “help-seeking” for conceptual clarity, the operational search parameters encompassed six Chinese lexical equivalents.

Text S2

Coding Analysis Framework for Weibo Posts

Social media datasets are multi-dimensional.[1] There is evidence that studies have categorized text content posted on social media to obtain more information.[2,3] Previous studies have particularly focused on four dimensions - Space, Content, Network, and Time - for extracting valuable information to enhance situational awareness and better disaster response.[1,4] For our analysis, the "Space" dimension was considered constant, as established earlier, with our sample area being specifically focused on mainland China. The data was encoded based on the remaining three dimensions: Content, Network, and Time. In our study, “Content”, which typically composed of theme and emotional attributes,[3] was categorized by different types of posts. The “Network” dimension, usually defined as “information exchange behaviors of various agents”, was subdivided in our study into two distinct sub-dimensions: “Responders”, referring to those who comment, and “Response Types”, which focused on the nature or substance of the comments. “Time” was divided into four distinct periods. Altogether, these four dimensions were represented through 20 specific items.

Details are explained as follows:

- Content — Post Types: 1) Help-seeking: Medicine-seeking or Targeting others; 2) Aids provision: Medicine donations and Offering suggestions; 3) Official guidance: reposting official guidance from, e.g., the China CDC; 4) Daily life recording: positive or negative posts commenting on users’ experiences or feelings about life with COVID-19.

- Network A — Responders: 1) Community level (e.g., individual / neighborhood / community service center, etc.); 2) Governmental: relevant government departments; 3) Market-based: channels for purchasing medication, including pharmacies, hospitals, online platforms.

- Network B — Response Types: 1) Tangible assistance or aid: community-based donations; obtainment of medicine from medical suppliers; 2) Information or advice: Guidance regarding medication or home remedies; Information regarding purchasing; 3) Emotional response: positive and negative.

- Time — Time Periods: 1) First week (December 8, 2022, to December 15, 2022); 2) Second week (December 16, 2022, to December 23, 2022); 3) Third week (December 24, 2022, to December 31, 2022); 4) Fourth week (January 1, 2023, to January 7, 2023).

Text S3

Analysis Plan

To enhance replicability, this study provided a detailed description of the analytical workflow. The study design involved multiple sequential steps, including data preparation, coding, text classification, indicator construction, clustering, and post-hoc analyses. Each step was described below:

1. Data preparation

We retrieved raw data from the Sina Weibo big data system covering the period December 8, 2022, to January 7, 2023, which corresponded to the earliest stage of China's reopening from the "Zero-COVID" policy.

- Keyword filtering: A set of predefined COVID-19–related keywords was used to capture posts about help-seeking, resource provision, and daily life experiences related to the pandemic.
- Initial retrieval: 199,790 posts were initially downloaded.
- Exclusion criteria: Posts originating from outside mainland China, with unverifiable IP addresses, or irrelevant to COVID-19 were excluded.
- Final dataset: 177,000 posts were retained for subsequent analysis.

This filtering ensured that the dataset captured community-level responses to the reopening, including expressions of need, guidance dissemination, and everyday coping strategies.

2. Coding procedure

A structured coding process was designed to generate a reliable labelled dataset for supervised learning.

- Pilot coding: A random subset of 2,000 posts was independently coded by multiple authors to test the coding rules. Discrepancies were discussed collectively and used to refine the coding analysis framework.
- Main coding: After refinement, a random subset of 10,000 posts was selected from the cleaned dataset. Two authors independently coded these posts, applying the finalized coding framework.
- Inter-coder reliability: Agreement exceeded 90%, indicating strong consistency.
- Consensus resolution: Remaining discrepancies were resolved through team consensus.

The final manually labelled dataset was then used to train and evaluate ML models for text classification.

3. Text classification

We implemented a BERT-based Chinese classifier to categorize posts into pre-specified categories.

- Cross-validation: Five-fold cross-validation was performed to identify the best-performing models.
- Prediction: The trained model assigned probability scores to each post, followed by a softmax function to determine the most likely label.
- Final categorization: All 177,000 posts were automatically categorized into one of four dimensions, which were represented through 20 specific items.

This step ensured scalability, allowing for the classification of a large dataset while maintaining consistency with human-coded examples.

4. Indicator framework construction

Based on previous literature and theoretical models of community resilience, we developed a framework of 13 indicators, grouped into five conceptual domains:

- Collective efficacy (X_1-X_2): measures of coordinated community actions and mutual assistance.
- Community emotional support (X_3-X_7): expressions of empathy, encouragement, and shared emotional coping.
- Social capital support (X_8-X_9): resource mobilization through networks, trust, and reciprocity.
- Community information sharing (X_{10}): dissemination and amplification of guidance, advice, and preventive measures.
- Rapid response performance of community networks (X_{11}-X_{13}): timeliness and effectiveness in addressing urgent needs.

These indicators were standardized before analysis to ensure comparability across dimensions.

5. Resilience evaluation via clustering

Community resilience levels were identified through K-means clustering applied to the 13 indicators.

- Standardization: All features were z-score standardized to remove scale bias.
- Clustering: K-means was applied with different values of K (3-7).
- Model diagnostics:
  - Silhouette coefficient: Evaluated cohesion and separation. The coefficient

peaked at K=4 (0.7003), higher than K=3 (0.6894), K=5 (0.6968), K=6 (0.6723), and K=7 (0.6761).

• Elbow method: Showed a clear inflection at K=4, after which reductions in WCSS diminished (Appendix H: Figure S3).

• Visualization: PCA-based scatterplots (Appendix G: Figure S2) confirmed clusters were compact and largely spherical, with limited overlap.

- Final classification: Four resilience levels were identified: High, Medium-high, Medium-low, and Low.

6. Quantitative descriptive analysis

To interpret resilience patterns, we conducted descriptive analyses at both temporal and regional levels.

- Temporal dynamics: We examined the relationship between help-seeking posts and newly confirmed COVID-19 cases. Help-seeking surged and peaked earlier than confirmed cases, suggesting posts served as a proxy for real-time community demand.
- Regional heterogeneity: We compared resilience levels across regions. Eastern provinces showed the highest resilience, while northeastern provinces displayed the lowest resilience.

7. Indicator importance analysis

To assess the relative contribution of each indicator to resilience levels, we applied a Random Forest (RF) model and computed SHAP values.

- Interpretability: SHAP values ranked indicators by their marginal contribution to cluster assignment.

Table S1. English Keywords.

Help-related terms	Medicine-related terms
Help<br>Help-seeking	Ibuprofen, Acetaminophen, Ambroxol, Dextromethorphan, Aspirin, Chlorpheniramine, Loratadine, Cetirizine, Xylometazoline, Bromhexine, Guaifenesin, Acetylcysteine, Pholcodine

Table S2. Chinese Keywords.

Help-related terms	Medicine-related terms
求助，求药，<br>帮助，救助，给药，送药	布洛芬，对乙酰氨基酚，氨溴索，右美沙芬，阿司匹林，氯苯那敏，氯雷他定，西替利嗪，赛洛唑啉，溴己新，愈创甘油醚，乙酰半胱氨酸，福尔可定

Table S3. Coding Analysis Framework for Weibo Posts.

Item Numbers	Themes	Definitions	Examples
	● **Content**	**Post Types**	
	**Help-seeking**	Posts seeking help with medication or other issues	--
Item 1	Medicine-seeking	Posts regarding sourcing medicine, whether from medical sources or individuals	“Does anyone have an extra ibuprofen I can get? HELP.”
Item 2	Targeting others	Posts seeking help or advice on topics other than medication, such as assistance with antigen tests, or pulse oximeters	“Got a fever ! Can anyone help me find the best place for a rapid COVID antigen test?”
	**Aids provision**	Posts advising others regarding the possibility of medication donation or providing other advisory support	--
Item 3	Medicine donations	Posts about potentially donatable medication in the possession of the author	“I have extra ibuprofen to send in Haidian District. If you need it, please comment below this post!”
Item 4	Offering suggestions	Posts sharing experience and substantial advice regarding dealing with COVID-19	“If you have no medicine, drinking hot water and ginger soup can reduce COVID fever and save your pain.”
Item 5	**Official guidance**	Reposting official guidance from, e.g., the Chinese Center for Disease Control and Prevention (China CDC)	For anyone who needs help could check “CDC Guidelines of Community Health Management for COVID-19 Positive cases”
	**Daily life recording**	Posts commenting on users’ experiences or feelings about life with COVID-19	
Item 6	Positive	Positive content: e.g.,	“Big thanks to my

Item Numbers	Themes	Definitions	Examples
		encouragement, gratitude, excitement	neighbor for sharing ambroxol with me! She really saved my life."
Item 7	Negative	Negative content: e.g., complaints, anger, sadness	"After being infected, I felt pain everywhere and hate the COVID! No can help me!"
●	● **Network A**	**Responders**	--
Item 8	**Community level**	Community members, neighbours, local residents, community service centers (e.g., community healthcare center, residents' committees, owners' committees)	Residents' Committee of Beijing Vank Community.
Item 9	**Governmental**	Relevant government departments	Municipal Health Commission of sample area.
Item 10	**Market-based**	Channels for purchasing medication, including pharmacies, hospitals, online platforms	YF Online Pharmacy.
●	● **Network B**	**Response Types**	
	**Tangible assistance or aid**	Providing medication to the target user	--
Item 11	Community-based donations	Personal interactions through delivery, a transaction or delivery of the medication	"I live near you and can deliver medicine to you tonight."
Item 12	Medical suppliers	Obtaining medicine from community healthcare centers, hospitals or other health institutions	"Hi, our online pharmacy sales will contact you soon."
	**Information or advice**	Suggestions and advice provided in the comments section regarding the proper use of medication	--
Item 13	Guidance regarding medication or home remedies	Comments regarding the correct use of a particular medication or potentially useful home remedies	"Remember don't take Chinese medicine and Western medicine

Item Numbers	Themes	Definitions	Examples
			together."
Item 14	Information regarding purchasing	Advising the target user about where he or she can purchase medication	"Just got cough medicine at the children's hospital, they still have stock. If you need some, hurry over!"
	**Emotional response**	Emotional content	
Item 15	Positive	Positive content: e.g., encouragement, gratitude, excitement	"Thank you very much for your help, fell better after taking medicine!"
Item 16	Negative	Negative content: e.g., complaints, anger, sadness	"I really can't accept that the antipyretics were so expensive!"
●	● **Time**	**Time Periods**	
Item 17	**First week**	Posts published between December 8th, 2022 and December 15th, 2022	--
Item 18	**Second week**	Posts published between December 16th, 2022 and December 23rd, 2022	--
Item 19	**Third week**	Posts published between December 24th, 2022 and December 31st, 2022	--
Item 20	**Fourth week**	Posts published between January 1st, 2023 and January 7th, 2023	--

Table S4. Performance Evaluation of Each Text Classification Task.

Categories	AUC (95% CI)	ACC (95% CI)	Precision (95% CI)	Recall (95% CI)	F1 (95% CI)
Medicine-seeking	0.9115 (0.9073, 0.9162)	0.8385 (0.8255, 0.8389)	0.7708 (0.7515, 0.7718)	0.8369 (0.8207, 0.8394)	0.8025 (0.7869, 0.8032)
Targeting others	0.7948 (0.7811, 0.8335)	0.9707 (0.9700, 0.9755)	0.5775 (0.5770, 0.8112)	0.3624 (0.1990, 0.3635)	0.4454 (0.3081, 0.4485)
Medicine donations	0.9537 (0.9509, 0.9603)	0.9294 (0.9157, 0.9298)	0.8471 (0.7789, 0.8479)	0.7624 (0.7623, 0.8011)	0.8026 (0.7779, 0.8043)
Offering suggestions	0.9435 (0.9353, 0.9471)	0.9211 (0.9125, 0.9235)	0.7866 (0.7534, 0.7875)	0.7776 (0.7491, 0.7849)	0.7821 (0.7559, 0.7841)
Official guidance	0.9462 (0.9396, 0.9486)	0.9136 (0.9042, 0.9143)	0.7474 (0.7175, 0.7563)	0.7775 (0.7373, 0.7786)	0.7621 (0.7333, 0.7662)
Daily life recording (Positive)	0.7840 (0.7839, 0.8051)	0.7090 (0.7065, 0.7554)	0.5574 (0.5563, 0.6768)	0.7033 (0.5153, 0.7064)	0.6219 (0.5752, 0.6280)
Daily life recording (Negative)	0.8575 (0.8495, 0.8646)	0.8538 (0.8526, 0.8812)	0.4863 (0.4850, 0.6311)	0.6023 (0.3490, 0.6029)	0.5381 (0.4350, 0.5482)
Community level	0.9132 (0.9114, 0.9215)	0.8475 (0.8387, 0.8515)	0.8437 (0.8430, 0.8788)	0.9171 (0.8643, 0.9182)	0.8789 (0.8661, 0.8860)
Governmental	0.9243 (0.8772, 0.9368)	0.9936 (0.9895, 0.9941)	0.8889 (0.7142, 0.8896)	0.6713 (0.5481, 0.6939)	0.7649 (0.6169, 0.7685)
Market-based	0.9004 (0.8923, 0.9087)	0.9124 (0.9037, 0.9145)	0.6115 (0.5681, 0.6193)	0.5906 (0.5506, 0.6040)	0.6009 (0.5658, 0.6080)
Community-based donations	0.8383 (0.8382, 0.9213)	0.8072 (0.8056, 0.8550)	0.6585 (0.6574, 0.7612)	0.7162 (0.7143, 0.8058)	0.6861 (0.6823, 0.7783)
Medical suppliers	0.8221 (0.8220, 0.9560)	0.9158 (0.9157, 0.9866)	0.3780 (0.3543, 0.6667)	0.4697 (0.0858, 0.4758)	0.4189 (0.1388, 0.4275)
Guidance regarding medication or home remedies	0.8836 (0.8723, 0.8891)	0.8781 (0.8656, 0.8793)	0.6176 (0.6155, 0.6882)	0.7354 (0.4613, 0.7385)	0.6714 (0.5417, 0.6752)
Information regarding purchasing	0.8692 (0.8691, 0.9165)	0.9803 (0.9793, 0.9866)	0.4286 (0.4280, 0.7719)	0.3519 (0.1608, 0.3577)	0.3864 (0.2500, 0.3869)
Emotional response (Positive)	0.9139 (0.8317, 0.9140)	0.8516 (0.7766, 0.8519)	0.7479 (0.6552, 0.7488)	0.8070 (0.5098, 0.8071)	0.7763 (0.5749, 0.7782)
Emotional response (Negative)	0.9224 (0.8058, 0.9225)	0.9801 (0.9312, 0.9809)	0.3879 (0.3856, 0.6000)	0.4384 (0.0761, 0.4396)	0.4116 (0.1295, 0.4166)

AUC – area under the curve, AUC – accuracy, CI – confidence interval.

Table S5. Regional Distribution of Mainland China.

Regions *	Provinces / Municipalities / Autonomous regions
Eastern	Beijing / Tianjin / Hebei / Shanghai / Jiangsu / Zhejiang / Fujian / Shandong / Guangdong / Hainan
Central	Shanxi / Anhui / Jiangxi / Henan / Hubei / Hunan
Western	Inner Mongolia / Guangxi / Chongqing / Sichuan / Guizhou / Yunnan / Xizang / Shaanxi / Gansu / Qinghai / Ningxia / Xinjiang
Northeastern	Liaoning / Jilin / Heilongjiang

* For the classification standards of regions in mainland China, we refer to the Chinese National Bureau of Statistics (https://www.stats.gov.cn).

Table S6. Mean SHAP Value of Indicators.

**Indicators**	**Mean SHAP Value**
Help-seeking response efficacy (X_1)	0.0035
Efficacy of performance of altruistic response (X_2)	0.0101
Sentiment response associated with help-seeking (X_3)	0.0024
Sentiment response associated with altruistic posts (X_4)	0.0021
Sentiment response to the sharing of official information (X_5)	0.0022
Sentiment response associated with recording positive posts (X_6)	0.0036
Sentiment response associated with recording negative posts (X_7)	0.0024
Tangible aid engagement (X_8)	0.0051
Intangible aid engagement (X_9)	0.0024
Interaction relating to repost/share official information (X_{10})	0.0025
Rapid aid reaction (X_{11})	0.0035
Rapid performance of altruism (X_{12})	0.0044
Rapid sharing of official information as a reaction (X_{13})	0.0017

Table S7. Results of Sensitivity Analysis.

	RF_Rank	XGBoost_Mean SHAP Value	XGBoost_Relative Contribution (%) *	XGBoost_Rank
X_2	1	0.8027	20.43	1
X_8	2	0.0511	1.30	13
X_{12}	3	0.3006	7.65	5
X_6	4	0.1848	4.70	9
X_1	5	0.6124	15.59	2
X_{11}	6	0.4134	10.52	3
X_{10}	7	0.2988	7.61	6
X_3	8	0.3626	9.23	4
X_9	9	0.2910	7.41	7
X_7	10	0.1898	4.83	8
X_5	11	0.1656	4.22	10
X_4	12	0.1625	4.14	11
X_{13}	13	0.0929	2.36	12

RF – random forest, XGBoost – extreme gradient boosting, X_1 – Help-seeking response efficacy, X_2 – Efficacy of performance of altruistic response, X_3 – Sentiment response associated with help-seeking, X_4 – Sentiment response associated with altruistic posts, X_5 – Sentiment response to the sharing of official information, X_6 – Sentiment response associated with recording positive posts, X_7 – Sentiment response associated with recording negative posts, X_8 – Tangible aid engagement, X_9 – Intangible aid engagement, X_{10} – Interaction relating to repost/share official information, X_{11} – Rapid aid reaction, X_{12} – Rapid performance of altruism, X_{13} – Rapid sharing of official information as a reaction.

* Relative contribution was calculated by dividing each indicator's mean SHAP value by the sum of SHAP values across all 13 indicators.

Appendix C

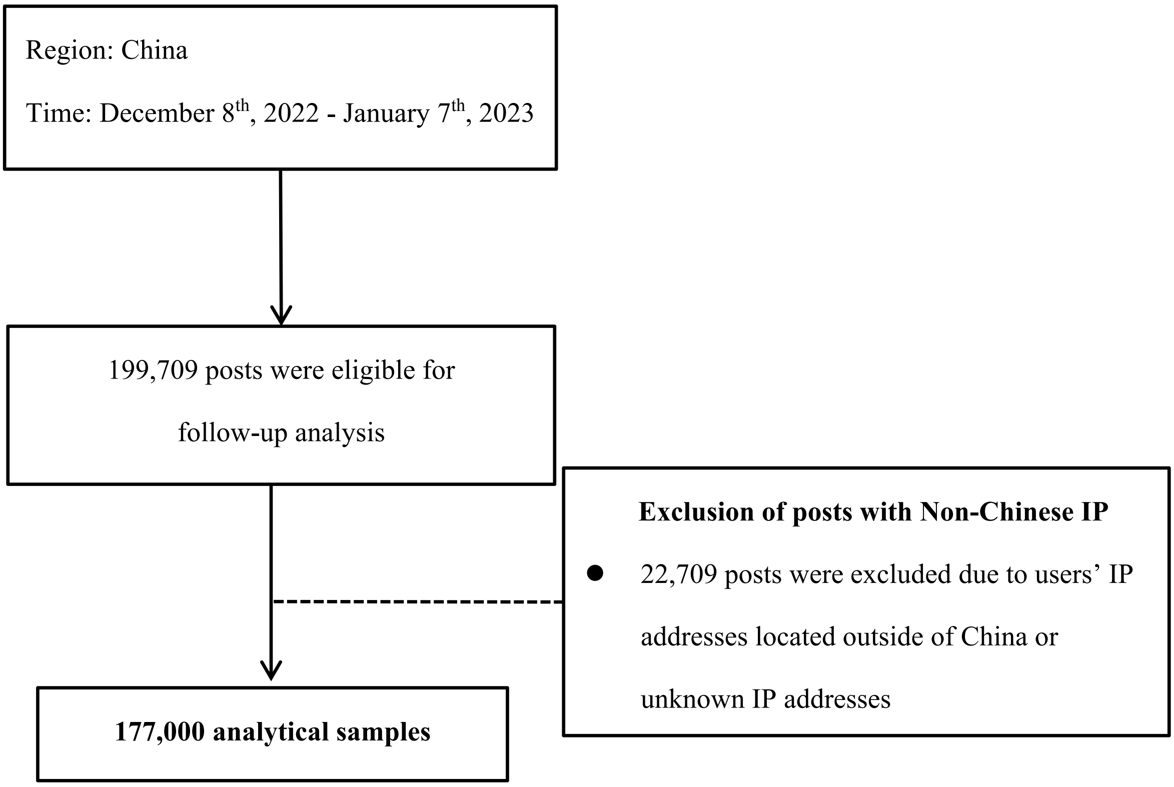

Figure S1. Flowchart of Dataset Combination.

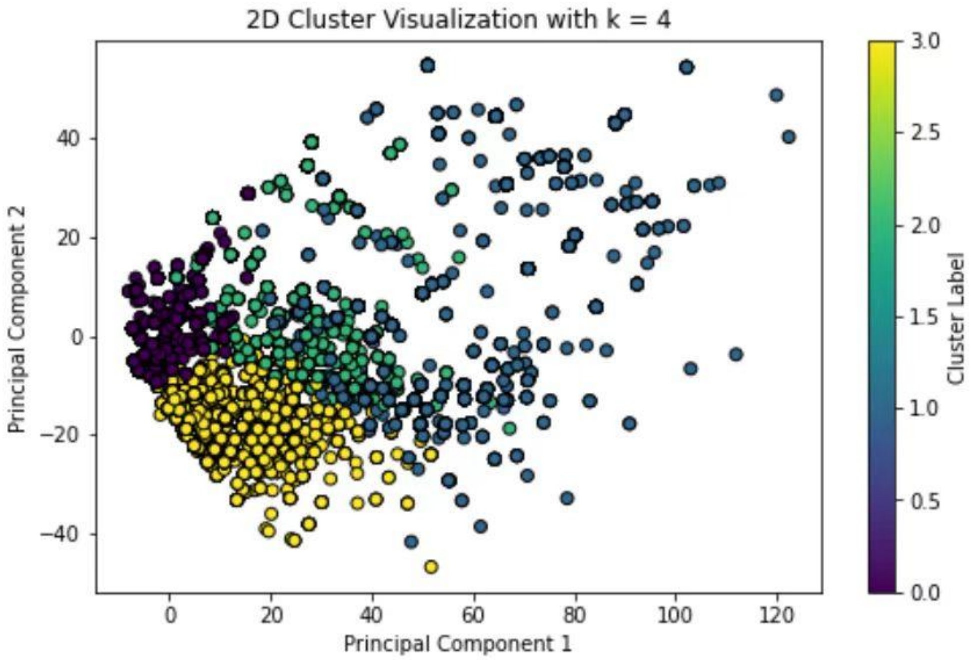

Figure S2. PCA-based Two-dimensional Visualization of K=4 Clusters.

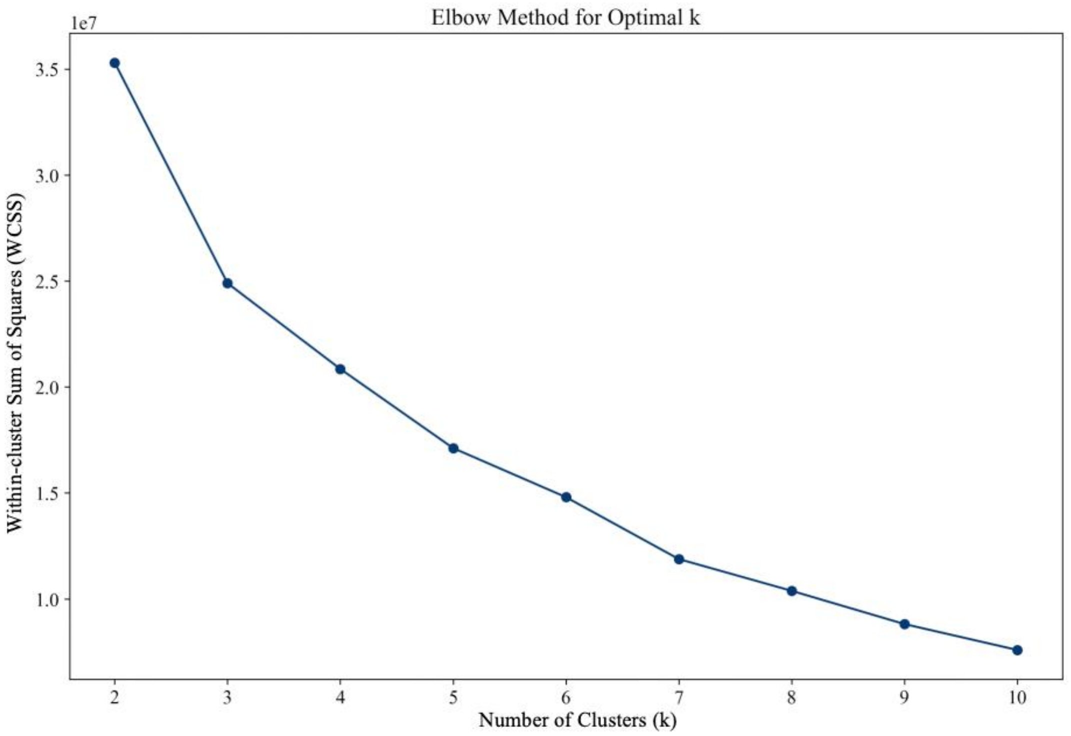

Figure S3. Elbow Method for Determining the Optimal Number of Clusters.

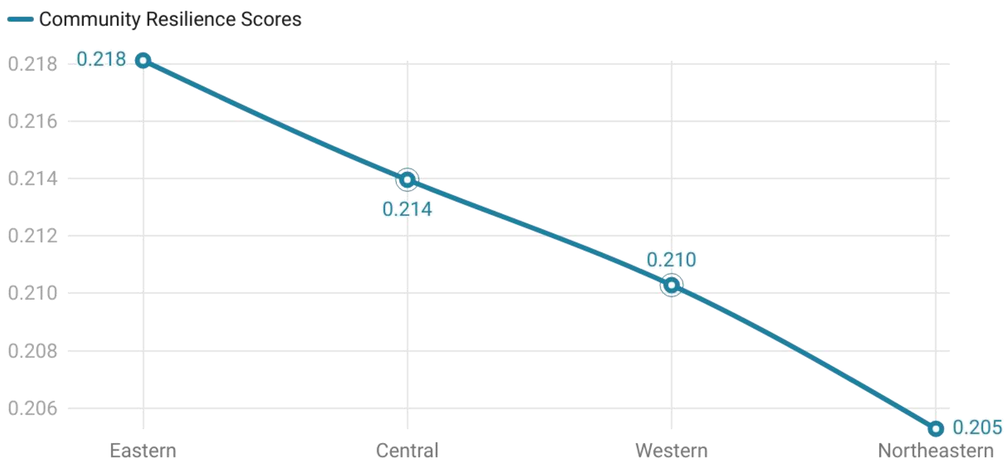

Figure S4. Community Resilience Scores Across Four Regions.

References

1. Wang Z, Ye X. Social media analytics for natural disaster management. *Int J Geogr Inf Sci*. 2018;32(1):49-72. https://doi.org/10.1080/13658816.2017.1367003
2. Qian Y, Ni Z, Zheng H, Liu Z, Ma F. Factors influencing users' post replying behavior in a senior online community: An empirical investigation. *Telemat Inform*. 2023;77:101926. https://doi.org/https://doi.org/10.1016/j.tele.2022.101926
3. Zhou B, Miao R, Jiang D, Zhang L. Can people hear others' crying?: A computational analysis of help-seeking on Weibo during COVID-19 outbreak in China. *Inf Process Manag*. 2022;59(5):102997. https://doi.org/https://doi.org/10.1016/j.ipm.2022.102997
4. Lai C-H, She B, Ye X. Unpacking the Network Processes and Outcomes of Online and Offline Humanitarian Collaboration. *Communic Res*. 2015;46(1):88-116. https://doi.org/10.1177/0093650215616862
